# Early Effects of Modern Radiotherapy for Lung Cancer on Endothelial Damage and Myocardial Fibrosis: A Prospective Single-Center Study

**DOI:** 10.3390/ijms25126705

**Published:** 2024-06-18

**Authors:** Grzegorz Sławiński, Maja Hawryszko, Zofia Lasocka-Koriat, Anna Romanowska, Kamil Myszczyński, Anna Wrona, Agata Ogłoza, Ludmiła Daniłowicz-Szymanowicz, Ewa Lewicka

**Affiliations:** 1 Department of Cardiology and Electrotherapy, Faculty of Medicine, Medical University of Gdańsk, 80-210 Gdansk, Poland; gslawinski@gumed.edu.pl (G.S.); ludwik@gumed.edu.pl (L.D.-S.); elew@gumed.edu.pl (E.L.); 2 1st Department of Cardiology, Faculty of Medicine, Medical University of Gdańsk, 80-210 Gdansk, Poland; zofia.lasocka@gumed.edu.pl; 3 Department of Oncology & Radiotherapy, Faculty of Medicine, Medical University of Gdańsk, 80-210 Gdansk, Poland; ankapoplawska@gmail.com (A.R.); wronania@gmail.com (A.W.); 4 Centre of Biostatistics and Bioinformatics Analysis, Medical University of Gdansk, 80-210 Gdansk, Poland; kamil.myszczynski@gmail.com; 5 Department of Haematology & Transplantology, Faculty of Medicine, Medical University of Gdańsk, 80-210 Gdansk, Poland; aogloza@uck.gda.pl

**Keywords:** radiotherapy, lung cancer, endothelial dysfunction, myocardial fibrosis, cardio-oncology

## Abstract

Radiotherapy (RT) may have a cardiotoxic effect on the heart and cardiovascular system. Postulated mechanisms mediating these complications include vascular endothelium damage and myocardial fibrosis. The aim of our study was to assess endothelial damage and myocardial fibrosis in the early period after RT on the basis of cardiac biomarkers and in relation to the radiation dose applied to individual heart structures in patients treated for non-small-cell lung cancer. This single-center prospective study included consecutive patients with lung cancer (LC) who were referred for treatment with radiochemotherapy (study group) or chemotherapy (control group). The study protocol included performing an echocardiographic examination, a standard ECG examination, and collecting blood samples for laboratory tests before starting treatment for lung cancer in the first week after completing RT (after four cycles of chemotherapy in the control group) and after 12 weeks from the end of treatment. The study included 23 patients in the study group and 20 patients in the control group. Compared to the baseline values, there was a significant increase in total cholesterol concentration in the study group immediately after the end of RT, which persisted for three months after the end of therapy. After taking into account the use of statins in the analysis, it was found that an increase in total cholesterol concentration after oncological treatment was observed only among patients who did not use statins. Taking into account the assessment of myocardial fibrosis markers, there were no significant changes in the concentration of matrix metallopeptidase 9 (MMP-9) and tissue inhibitors of metalloproteinases 1 (TIMP-1) in the study group. In patients treated with radiochemotherapy, there was a significant increase in the concentration of intercellular adhesion molecule 1 (ICAM-1) immediately after RT, when compared to the baseline. After taking into account the use of statins, an increase in ICAM-1 concentration immediately after RT was observed only in patients who did not use statins. There was also a significant correlation between the radiation dose received by the left anterior descending coronary artery (LAD) and left circumferential coronary artery, and vascular cell adhesion protein 1 (VCAM-1) concentration measured at three months after the end of RT. Immediately after completion of radiotherapy, a significant increase in the level of ICAM-1 is observed indicating endothelial damage. The radiation dose to coronary arteries should be minimized, as it correlates with the concentration of VCAM-1. The use of statins may prevent the increase in total cholesterol and ICAM-1 concentration after irradiation for lung cancer; however, further studies designed for this specific purpose are necessary to confirm the effectiveness of statins in this area.

## 1. Introduction

Radiotherapy (RT) is a recognized method for the treatment of many cancers. However, it has been indicated that RT may have a cardiotoxic effect on the heart and cardiovascular system. Long-term complications of RT are well known, such as coronary artery disease (CAD), valvular heart disease (VHD), heart failure (HF), cardiomyopathy, arrhythmias, and pericardial syndromes [1]. The postulated mechanisms mediating these complications include vascular endothelium damage and myocardial fibrosis [2,3]. Diagnostics use imaging tests (magnetic resonance imaging, computed tomography, echocardiography), electrocardiography, and laboratory markers. It is suggested that fibrosis after radiotherapy occurs more frequently in certain segments of the myocardium (septal and apical regions), and the radiation dosage used in radiotherapy is of key importance [4]. Echocardiography suffers from some limitations, such as poor acoustic windows or intra/inter-reader variability [5,6]. In turn, in the case of computed tomography, ionizing radiation is used, which must be considered for serial follow-up and in the cases involving young patients. In the case of magnetic resonance imaging, the limitations are price, availability, and several contraindications to the examination (for instance, claustrophobia or magnetic-resonance-incompatible implants) [7]. For this reason, for a long time, researchers have been focusing on identifying laboratory markers that could be used in the early diagnosis of complications of radiotherapy, such as myocardial fibrosis or endothelial dysfunction. The use of amifostine, ACE inhibitors, statins, colchicine, sestrin2, fibrates, pentoxifylline, black grape juice, and thalidomide has been studied in the treatment or prevention of radiation-induced myocardial fibrosis. Currently, there is no consensus recommending the use of specific pharmacotherapy in these patients [8]. The aim of our study was to assess endothelial damage and myocardial fibrosis in the early period after RT on the basis of cardiac biomarkers and in relation to the radiation dose applied to individual heart structures in patients treated for non-small-cell lung cancer (NSCLC).

## 2. Results

The study included 43 patients in a mean age of 64.9 ± 8.1 years, and 25 were men (58.1%). There were 23 patients in the study group and 20 in the control group. The planned three follow-up visits were completed by 60% of patients. During the follow-up, eight patients (19%) withdrew from the study because of their poor general condition, and nine patients (21%) died.

The baseline characteristics of the studied population are shown in Table 1. The most common NSCLC was adenocarcinoma (46.5%) and squamous cell carcinoma (46.5%). The most common comorbidity was arterial hypertension. Nearly all patients (91%) were active or former smokers. Chemotherapeutic agents used were cisplatin (*n* = 19), pemetrexed (*n* = 17), and carboplatin (*n* = 15). There were no differences in chemotherapy regimens between the groups. The median dose of radiation to the whole heart was 9.7 Gy, with no significant differences between the patients with left-sided and right-sided lung cancer (9.7 Gy vs. 9.8 Gy, respectively; *p* = 0.95).

Patients included in the study showed preserved LV and RV systolic function (Supplementary Tables S1 and S2). The mean LV ejection fraction obtained using three-dimensional echocardiography (3D LVEF) was 53.8% ± 1.5%, and RV ejection fraction (3D RVEF) was 52.0% ± 7.0%. Baseline BNP serum concentration was normal (66.6 ± 63.8 pg/mL), and there were no significant changes in BNP levels during follow-up in both groups (Table 2).

**Figure 1:**
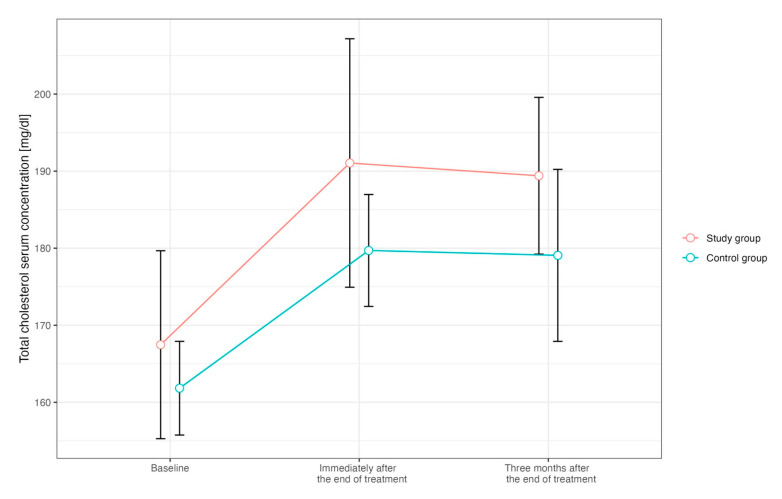
Changes in total cholesterol concentration over time in patients from the study group (red line) and control group (blue line).

Compared to the baseline values, there was a significant increase in total cholesterol concentration in the study and control group immediately after the end of RT, which persisted for three months after the end of therapy (Figure 1). Only in patients from the study group, there was also a significant increase in LDL-cholesterol concentration when examined immediately after the end of RT (119.4 vs. 107.2 mg/dL, *p* = 0.048). In total, 19 out of all patients used a statin. After taking into account the use of statins in the analysis, it was found that an increase in total cholesterol concentration after oncological treatment was observed only among patients who did not use statins: initially, 186.8 mg/dL; immediately at the end of treatment, 206.2 mg/dL (*p* = 0.01); and three months after the end of treatment, 199.5 mg/dL (*p* = 0.005 compared to baseline).

**Figure 2:**
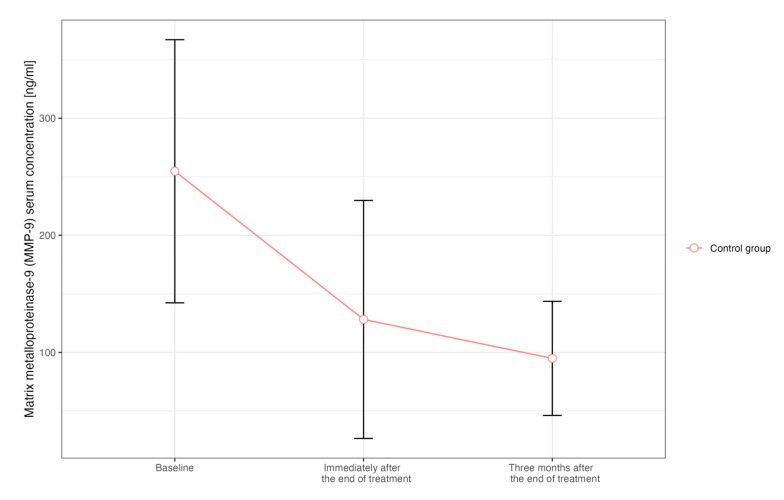
Change in the MMP-9 concentrations over time in patients from the control group (treated exclusively with chemotherapy).

Taking into account the assessment of myocardial fibrosis markers, there were no significant changes in the concentration of MMP-9 and TIMP-1 in the study group. However, a significant decrease in MMP-9 concentration over time was confirmed among patients from the control group (Figure 2).

In patients treated with radiochemotherapy, there was a significant increase in the concentration of ICAM-1, immediately after RT, when compared to the baseline (1507 ng/mL vs. 1272 ng/mL, *p* = 0.008). This change was not observed in patients who did not receive radiotherapy. After taking into account the use of statins, an increase in ICAM-1 concentration immediately after RT was observed only in patients who did not use statins (555.0 ng/mL vs. 711.0 ng/mL at baseline and after treatment, respectively; *p* = 0.039), while in patients using statins, no significant increase in ICAM-1 concentration was found (500.0 ng/mL vs. 647.0 ng/mL at baseline and after treatment, respectively; *p* = 0.26).

**Figure 3:**
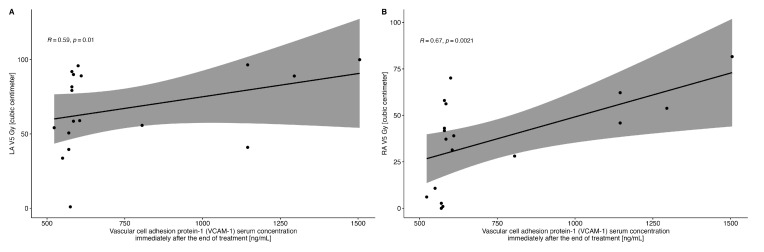
Correlation between the VCAM-1 concentration measured immediately after completion of radiotherapy and the LA V5Gy (Panel (**A**)) and RA V5Gy (Panel (**B**)) volumes.

There was a significant correlation between the LA V5Gy volume, as well as RA V5Gy volume and VCAM-1 concentration measured immediately after the completion of RT (Figure 3).

**Figure 4:**
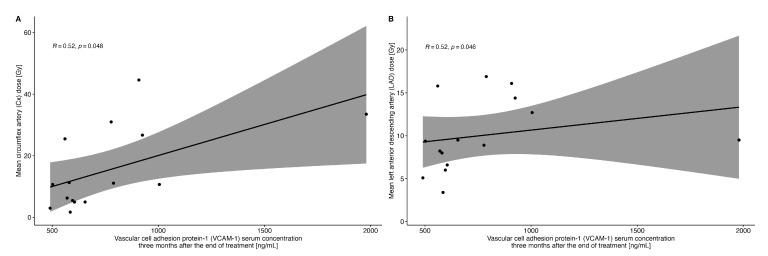
Correlation between VCAM-1 concentration three months after radiotherapy and the radiation dose received by the circumflex artery (Panel (**A**)) and left anterior descending artery (Panel (**B**)).

There was also a significant correlation between the radiation dose received using LAD (r = 0.52; *p* = 0.046) and Cx (r = 0.52; *p* = 0.048) and the VCAM-1 concentration measured at three months after the end of RT (Figure 4).

## 3. Methods

This single-center prospective study performed between 15 November 2020 and 28 March 2022 included consecutive patients with lung cancer (LC) who were referred for treatment with radiochemotherapy (study group) or chemotherapy (control group). The study was conducted at the Department of Cardiology and Electrotherapy of the Medical University of Gdańsk in Poland in cooperation with the Department of Oncology and Radiotherapy at the same institution.

The inclusion criteria were:

- Age over 18 years.
- Histopathological diagnosis of lung cancer.
- Future planned treatment with the use of radiochemotherapy or chemotherapy.
- Written consent of the patient to participate in the study.

The exclusion criteria were:

- Future planned or previous surgical treatment of lung cancer.
- History of potentially cardiotoxic oncological treatment (radiotherapy, chemotherapy, or immunotherapy).
- Severe functional impairment (assessed using the Karnofsky Performance Scale).
- No written consent to participate in the study.

The study protocol included performing an echocardiographic examination, a standard ECG examination, and collecting blood samples for laboratory tests before starting treatment for lung cancer in the first week after completing RT (after 4 cycles of chemotherapy in the control group) and after 12 weeks from the end of treatment. Blood samples were collected at each stage of the study to measure the serum concentration of endothelial damage biomarkers, namely vascular cell adhesion protein 1 (VCAM-1) and intercellular adhesion molecule 1 (ICAM-1), and biomarkers of myocardial fibrosis, namely matrix metallopeptidase 9 (MMP-9) and tissue inhibitor of metalloproteinases 1 (TIMP-1). In addition, the serum concentration of high-sensitivity troponin I (hs-TnI), cardiac isoenzyme creatine kinase (CK-MB), B-type natriuretic peptide (BNP), hemoglobin, creatinine, total cholesterol, and LDL cholesterol were also determined. The VCAM-1, ICAM-1, MMP-9, and TIMP-1 concentrations were assessed using the ELISA (Enzyme-Linked Immunosorbent Assay) tests.

Data on medical history and treatment were collected from the patients’ medical charts. Echocardiographic examinations were performed at each stage using the same protocol, personnel, and equipment: a Vivid S95 (General Electric Medical Health, Chicago, IL, USA). Examinations were performed using parasternal, apical, and subcostal views, including dedicated right ventricular (RV) views. Cine loops from three standard apical views (4-, 3-, and 2-chamber) were recorded for offline analysis (EchoPac 201, GE Healthcare, Milwaukee, WI, USA). All measurements and calculations were obtained according to the recommendations for cardiac chamber quantification [9].

RT planning and treatment were conducted in accordance with the local institutional protocol. The planning target volume (PTV) dose was mostly 66 Gy, delivered in 30 daily fractions. In most cases, four-dimensional scans (4D CT) were used for planning purposes, and RT was delivered with dynamic techniques. All patients were irradiated with 6 MeV photon beams using a linear accelerator (TrueBeam^®^ SN1403 accelerator, Varian Medical Systems Inc., Palo Alto, CA, USA). Individual structures of the heart: left atrium (LA), right atrium (RA), RV, left ventricle (LV), pericardium, left anterior descending coronary artery (LAD), left circumferential coronary artery (Cx), and right coronary artery (RCA) were contoured on the ‘average’ scan set of the 4D CT or on the non-contrast enhancement scan of conventional planning CT on the basis of the existing atlases by two radiation oncologists [10]. Dosimetric analysis was performed using dose distribution data of the Eclipse^©^ Radiotherapy Planning System (Varian Medical Systems Inc.^©^, Palo Alto, CA, USA). Dose-volume histograms were generated for organs at risk. Calculated parameters included the mean heart dose and the mean doses for the following structures: pericardium, RV, LV, RA, LA, LAD, Cx, and RCA, the heart volume receiving 5 Gy (V5Gy), 25 Gy (V25Gy), and 30 Gy (V30 Gy), and the RV, LV, RA, and the LA volume receiving 5, 25, and 30 Gy.

### Statistical Analysis

Continuous variables were expressed as mean ± SD if normally distributed, or median if not normally distributed. For continuous variables, normal distribution was tested using the Shapiro–Wilk test. Categorical data were expressed as numbers and percentages. Continuous variables were compared using the independent-sample parametric (unpaired Student *t*) or nonparametric (Mann–Whitney *U* test, Kruskal–Wallis test by ranks) tests. Categorical variables were compared using the chi-square test or the Fisher exact test, where appropriate. Longitudinal data were compared using a paired *t*-test. Statistical significance values were corrected for multiple comparisons using the Benjamini–Hochberg procedure. Correlations between the concentrations of the assessed biomarkers and RT dosimetric parameters were assessed using Spearman’s rank correlation. Data were analyzed with the use of an R environment.

The study was approved by the Independent Bioethical Committee at the Medical University of Gdańsk (NKBBN/575/2020), and the study was carried out thanks to a grant from the Polish National Science Center-2020/04/X/NZ5/00387.

## 4. Discussion

Radiation-induced heart disease (RIHD) is the leading non-oncological cause of death in cancer patients. RIHD can occur both as an acute or a late complication, the latter usually 10–15 years after RT. Radiation-induced myocardial fibrosis (RIMF) is found in both acute and late manifestations of RIHD. Ultimately, RIMF can lead to the occurrence of heart failure, malignant ventricular arrhythmias, and sudden cardiac death. Heart failure may be limited to RV failure only, or diastolic LV failure only.

The pathogenesis of RIMF is not fully understood, but it is assumed that miRNAs, fibroblasts, transforming growth factor beta (TGF-β), and peroxisome proliferator-activated receptor (PPAR) alpha are involved in it. Due to the lack of complete insight into the pathogenesis of RIMF, effective treatment of this complication is difficult, and therefore the prevention of its occurrence is of primary importance. Three phases of RIMF development are distinguished [8]. In the first phase, which starts about six hours after radiotherapy, all layers of the myocardium and small blood vessels are affected by the inflammatory process, involving neutrophils and cytokine release such as monocyte chemotactic factor, tumor necrosis factor (TNF), and interleukins (IL-1, IL-6, and IL-8) [11,12]. The final effect of this phase is the transformation of monocytes into the M2 subset of macrophages that secrete a transforming growth factor β (TGF-β), which is responsible for the differentiation of fibroblasts into myofibroblasts [13]. In the second phase—about 2 days after irradiation—a gradual, mild fibrosis is observed, accompanied by damage to the capillary endothelium. The third phase occurs approximately 70 days after radiation, with extensive fibrosis resulting in a decrease in elasticity and distensibility [11,12]. In our study, we focused on assessing the severity of this late phase by assessing the concentrations of MMP-9 and TIMP-1 in patients undergoing RT for lung cancer.

RIMF diagnostics uses both biomarkers of myocardial damage (high-sensitivity troponin, BNP/NTproBNP) and multimodal imaging. Among the multimodal imaging methods, echocardiography, computed tomography, cardiac magnetic resonance, and nuclear cardiology are applicable [14]. Some authors point to the advantage of laboratory biomarkers over imaging tests in the initial asymptomatic phase of RIMF [15]. BNP concentrations do not become significantly elevated until 1 month after irradiation, and levels begin to decrease at 12 months after radiation in breast cancer patients [16,17]. In our patients, we confirmed that even during the first three months after the end of RT, there was no significant increase in BNP level.

Undoubtedly, an interesting finding is the significant increase in cholesterol concentration in patients after anticancer treatment, which persisted throughout the observation period. Additionally, LDL cholesterol concentration also increased significantly in the radiotherapy treatment group (119.4 vs. 107.2 mg/dL, *p* = 0.048). Furthermore, no significant increase in total cholesterol concentration was observed among patients using statins. These results correspond to the study by Boulet et al., who showed that the use of statin post-radiation therapy of the thorax, head, and neck was associated with a significant reduction in stroke, with a trend toward significantly reducing cardiovascular and cerebrovascular events [18]. Therefore, the use of statins should be considered in all patients treated with radiotherapy. However, randomized trials are needed to make such a recommendation.

It was shown that in healthy people, the serum concentration of MMP-9 is 29 ± 11 ng/mL. Among patients with malignant tumors (lung cancer, breast cancer), its concentration is significantly higher, up to 126–893 ng/mL [19]. This is in accordance with our results, as the mean concentration of MMP-9 was 259.12 ng/mL. Previous studies have confirmed that MMP-9 concentration correlates with cardiac remodeling in patients with heart failure, and could be useful in predicting left ventricular fibrosis [20]. In our study, there were no significant changes in myocardial fibrosis markers concentration over time among patients treated with radiotherapy. One explanation can be too short an observation period after RT—three months. Another factor is the small size of the group of patients treated with radiotherapy (*n* = 23), which means that despite the decrease in MMP-9 concentration compared to the baseline (283.88 ng/mL vs. 131.54), statistical significance was not achieved (*p* = 0.13). The fact that radiotherapy techniques have recently developed significantly, making them safer for the heart, should be also taken into consideration. Moreover, it was shown that the cancer itself and oncological treatment may affect the MMP-9 concentration. The expression and activity of MMP-9 are upregulated in NSCLC patients and are related to the pathologic type and clinical stage of NSCLC [21,22]. It was additionally found that MMP-9 has been implicated in cancer invasion and its metastases, and MMP-9 expression was associated with poor response to chemotherapy [23,24]. Therefore, the serum concentration of MMP-9 may decrease due to a good response to cancer treatment, which may mask myocardial fibrosis. For this reason, it seems that this marker is not useful for assessing myocardial fibrosis in cancer patients.

Endothelium damage due to radiotherapy results from its direct effect on the DNA, inflammatory cell activation, increased endothelial permeability, increased expression of cytokines (IL-1, IL-6, IL-8, TNF-alfa, TGF-beta), and adhesion molecules (VCAM-1, ICAM-1, E, and P-selectin), increased endothelium–neutrophil interaction, increased production of reactive oxygen species, increased mitochondrial damage and cell apoptosis [3,25]. In our study, we found a significant increase in ICAM-1 concentration in patients treated with radiotherapy, immediately after completion of RT, with a subsequent decrease to baseline values after three months. This may indicate temporary damage to the endothelium in the early period after radiotherapy. These data differ from those obtained in animal models, where ICAM-1 and VCAM-1 remained upregulated 20 weeks after irradiation [26]. No significant changes in ICAM-1 were observed among patients from the control group. A significant increase in ICAM-1 concentration was also not found in patients using statins, which may confirm their cardioprotective properties, which include protection against damage to the vascular endothelium. Importantly, we observed that the increase in VCAM-1 concentration correlated significantly with the radiation dose received by the coronary arteries (Cx and LAD) and with the LA V5Gy and RA V5Gy. The latter correlations are important, as it has been reported that concentrations of soluble VCAM-1, but not other inflammatory markers, are significantly associated with new-onset atrial fibrillation [27].

According to the Danish Breast Cancer Cooperative Group recommendations, a dose of 20 Gy per LAD should not be exceeded [28]. In our study patients, the mean radiation dose to the LAD was 9.6 ± 5.39 Gy, and yet it correlated with the change in VCAM-1 concentration, which confirms that there is probably no safe dose of radiation therapy that does not damage coronary arteries at the microvascular level.

## 5. Limitations of the Study

The main limitation of the study is the small size of the study group (43 patients in total), including those treated with radiotherapy (23 patients). Myocardial fibrosis was assessed only based on laboratory parameters, without the use of magnetic resonance imaging.

## 6. Conclusions

Modern radiotherapy for lung cancer is probably much less cardiotoxic in the early period three months after its completion, as indicated by the lack of changes in routine laboratory parameters such as hs-TnI and BNP. During this period, there are no changes in the concentration of TIMP-1 and MMP-9, and these biomarkers are not useful for monitoring the fibrosis processes in the cardiovascular system after RT. However, immediately after completion of radiotherapy, a significant increase in the level of ICAM-1 is observed, indicating endothelial damage. The radiation dose to coronary arteries should be minimized, as it correlates with the concentration of VCAM-1. The use of statins may prevent the increase in total cholesterol and ICAM-1 concentration after irradiation for lung cancer; however, further studies designed for this specific purpose are necessary to confirm the effectiveness of statins in this area.

## Figures and Tables

**Table 1:** Baseline characteristics of the study population.

Variable	All Patients (*n* = 43)	Study Group (*n* = 23)	Control Group (*n* = 20)	*p* Value
Age, years	64.9 ± 8.1	64.4 ± 8.6	65.5 ± 7.5	0.68
Males, *n* (%)	25 (58.1%)	13 (56.5%)	12 (60.0%)	0.82
Comorbidities, *n* (%):				
Arterial hypertension	26 (60.5%)	14 (60.1%)	12 (60.0%)	1.00
Coronary artery disease	7 (16.3%)	3 (13.0%)	4 (20.0%)	0.69
Heart failure	6 (13.9%)	3 (13.0%)	3 (15.0%)	0.32
Atrial fibrillation	14 (32.6%)	6 (26.1%)	8 (40.0%)	0.10
Hypercholesterolemia	7 (16.3%)	6 (26.1%)	1 (5.0%)	0.52
Diabetes	1 (2.3%)	1 (4.3%)	0	0.10
Chronic renal failure	5 (11.6%)	2 (8.7%)	3 (15.0%)	1.00
Chronic obstructive pulmonary disease	5 (11.6%)	2 (8.7%)	3 (15.0%)	1.00
Charlson Comorbidity Index, median	2 (0–6)	1 (0–3)	6 (2–7)	0.029
Smoking history, *n* (%):				1.00
Current	12 (27.9%)	6 (26.1%)	6 (30%)	
Former	27 (62.8%)	15 (65.2%)	12 (60%)	
Never	4 (9.3%)	2 (8.7%)	2 (10%)	
Number of pack years, *n*	37.5 ± 20.2	38.5 ± 20.6	36.3 ± 20.2	0.73
Histological type of lung cancer, *n* (%):				
Adenocarcinoma	20 (46.5%)	9 (39.1%)	11 (55.0%)	0.077
Squamous cell carcinoma	20 (46.5%)	14 (60.1%)	6 (30.0%)	
Small cell lung cancer	2 (4.7%)	0	2 (10.0%)	
Carcinoma not otherwise specified	1 (2.3%)	0	1 (5.0%)	
Chemotherapy drugs, *n* (%):				
Pemetrexed	17 (39.5%)	7 (30.4%)	10 (50%)	0.190
Cisplatin	19 (44.2%)	13 (56.5%)	16 (80%)	0.101
Carboplatin	15 (34.9%)	5 (21.7%)	10 (50%)	0.052
Paclitaxel	10 (23.2%)	5 (21.7%)	5 (25%)	0.798
Etoposide	4 (9.3%)	3 (13%)	1 (5%)	0.367
Vinorelbine	4 (9.3%)	2 (8.7%)	2 (10%)	0.084
Docetaxel	2 (4.6%)	0	2 (10%)	0.120
Radiotherapy characteristics				
RT duration, days	N/A	40 (40–40)	N/A	
Total RT dose, Gy		66 (63–66)		
Number of RT fractions		30 (30–30)		
RT technique:				
IMRT, *n* (%)		14 (60.9%)		
VMAT, *n* (%)		9 (39.1%)		
Heart dose, Gy		9.7 (6.7–13.2)		
Death, *n* (%):	9 (20.9%)	5 (21.7%)	4 (20%)	1.00

Abbreviations: IMRT—Intensity-modulated radiation therapy; N/A—not applicable; RT—radiotherapy; VMAT—Volumetric modulated arc therapy.

**Table 2:** Changes in the examined biomarkers concentrations and in other laboratory parameters in the compared groups of patients.

Variable	All Patients (*n* = 43)				
	Baseline	Immediately after Treatment	*p* Value	Three Months after Treatment	*p* Value (vs. Baseline)
BNP [pg/mL]	66.6 ± 63.8	78.6 ± 87.1	0.16	47.9 ± 38.6	0.71
hs-TnI [ng/mL]	0.003 ± 0.003	0.005 ± 0.014	0.16	0.003 ± 0.004	0.5
Creatinine [mg/dL]	0.80 ± 0.17	0.79 ± 0.17	0.48	0.87 ± 0.25	0.041
Total cholesterol [mg/dL]	171.1 ± 48.3	184.4 ± 40.2	0.003	184.4 ± 40.2	0.002
LDL [mg/dL]	105.6 ± 37.0	106.3 ± 41.0	0.55	109.6 ± 39.4	0.3
VCAM-1 [ng/mL]	805.21 ± 367.68	720.98 ± 247.63	0.87	774.45 ± 278.75	0.98
ICAM-1 [ng/mL]	1271.22 ± 427.28	1382.40 ± 492.28	0.021	1152.93 ± 403.71	0.37
MMP-9 [ng/mL]	259.12 ± 245.90	165.67 ± 144.60	0.005	120.28 ± 74.67	0.005
TIMP-1 [ng/mL]	195.47 ± 181.74	203.79 ± 281.45	0.41	117.77 ± 123.90	0.041
Variable	Study group (*n* = 23)				
BNP [pg/mL]	71.1 ± 56.4	87.6 ± 94.2	0.70	52.0 ± 38.7	0.20
hs-TnI [ng/mL]	0.003 ± 0.003	0.004 ± 0.004	0.17	0.004 ± 0.006	0.1
Creatinine [mg/dL]	0.84 ± 0.20	0.84 ± 0.20	0.28	0.93 ± 0.22	0.12
Total cholesterol [mg/dL]	177.3 ± 45.1	195.5 ± 60.7	0.022	189.4 ± 39.4	0.025
LDL [mg/dL]	107.2 ± 39.1	119.4 ± 47.5	0.048	107.6 ± 35.0	0.95
VCAM-1 [ng/mL]	765.17 ± 346.00	744.93 ± 304.18	0.68	768.53 ± 372.08	0.73
ICAM-1 [ng/mL]	1272.75 ± 397.71	1506.89 ± 545.72	0.008	1109.60 ± 369.23	0.28
MMP-9 [ng/mL]	283.88 ± 311.60	175.02 ± 176.26	0.21	131.54 ± 83.60	0.13
TIMP-1 [ng/mL]	203.09 ± 213.47	249.49 ± 373.56	0.63	130.29 ± 164.69	0.11
Variable	Control group (*n* = 20)				
BNP [pg/mL]	61.7 ± 72.3	69.1 ± 81.0	0.13	43.2 ± 39.4	0.53
hs-TnI [ng/mL]	0.003 ± 0.004	0.007 ± 0.019	0.46	0.001 ± 0.001	0.18
Creatinine [mg/dL]	0.75 ± 0.13	0.74 ± 0.12	0.88	0.81 ± 0.28	0.16
Total cholesterol [mg/dL]	164.1 ± 32.6	179.7 ± 30.0	0.037	179.1 ± 41.8	0.028
LDL [mg/dL]	103.7 ± 35.2	97.0 ± 26.5	0.19	111.6 ± 44.6	0.22
VCAM-1 [ng/mL]	851.25 ± 395.03	695.624 ± 175.11	0.49	780.79 ± 134.84	0.70
ICAM-1 [ng/mL]	1269.45 ± 469.49	1250.59 ± 403.29	0.80	1199.36 ± 446.90	0.88
MMP-9 [ng/mL]	230.64 ± 140.16	155.77 ± 105.93	0.004	108.22 ± 64.65	0.022
TIMP-1 [ng/mL]	186.70 ± 139.99	155.39 ± 123.63	0.47	104.34 ± 59.00	0.25

Abbreviations: BNP—B-type natriuretic peptide; ICAM-1—intracellular adhesion molecule-1; hs-TnI—high-sensitivity troponin I; LDL—low-density lipoprotein; MMP-9—matrix metalloproteinase-9; TIMP-1—tissue inhibitor of metalloprotease-1; VCAM-1—vascular cell adhesion molecule-1.

## Data Availability

The datasets used and/or analyzed during the current study are available from the corresponding author upon reasonable request.

## Supplementary Materials

The following supporting information can be downloaded at: https://www.mdpi.com/article/10.3390/ijms25126705/s1.
