# Supplementary material for: Early Effects of Modern Radiotherapy for Lung Cancer on Endothelial Damage and Myocardial Fibrosis: A Prospective Single-Center Study
Source: Int J Mol Sci. 2024 Jun 18;25(12):6705. doi: 10.3390/ijms25126705 (PMC11204135; doi:10.3390/ijms25126705)
Supplement: Supplementary file 1 [file ijms-25-06705-s001.zip › Supplementary Table S1.pdf]

Supplementary Table S1. Changes in the left heart dimensions and volumes in three consecutive echocardiographic examinations in the two groups of patients.

Variable	Baseline	After treatment	p value	Three months after treatment	p value (vs baseline)
All patients (n=43)					
LVEDV, ml	104.4±6.8	103.4±8.9	0.54	99.8±8.2	0.48
LVESV, ml	46.3±5.5	53.2±6.8	0.99	46.3±5.5	0.71
LVIDd, mm	48.2±1.9	46.3±1.2	0.98	48±1.9	0.18
LVIDs, mm	27.8±1.1	30.1±1.5	0.047	28.0±1.2	0.63
LA, mm	37.4±1.1	36.5±1.2	0.67	36.5±1.2	0.71
LAA, cm^2	20.2±1.0	19.8±1.3	0.60	21.1±1.5	0.11
3DLVEF, %	53.8±1.5	52.0±1.6	0.30	55.9±1.8	0.37
LV GLS, %	-16.7±0.5	-15.9±0.5	0.27	-16.8±0.6	0.08
Study group (n=23)					
LVEDV, ml	107.6±8.0	96.5±8.2	0.07	105.6±13.3	0.68
LVESV, ml	51.6±6.4	47.4±5.9	0.22	50.8±8.9	0.09
LVIDd, mm	47.1±1.0	46.8±1.3	0.53	51.9±2.9	0.008
LVIDs, mm	27.2±1.1	30.3±1.7	0.014	30.2±1.7	0.40
LA, mm	39.0±1.5	37.3±1.9	0.20	39.2±1.5	0.78
LAA, cm^2	22.0±1.6	21.4±2.4	0.91	24.4±2.4	0.50
3DLVEF, %	55.6±1.7	51.9±1.5	0.07	54.1±2.6	0.69
LV GLS, %	-17.2±0.5	-15.9±0.5	0.18	-16.0±0.7	0.03
Control group (n=20)					
LVEDV, ml	101±11.4	110.3±15.8	0.39	93.5±9.3	0.11
LVESV, ml	52.0±9.6	59.0±12.3	0.23	41.5±6.2	0.25
LVIDd, mm	49.5±4.0	45.8±2.2	0.55	43.2±1.8	0.46
LVIDs, mm	28.5±1.9	29.9±2.6	0.60	25.2±1.3	0.69
LA, mm	35.7±1.4	35.6±1.4	0.53	33.3±1.6	0.92
LAA, cm^2	18.1±1.0	18.2±0.9	0.37	17.6±1.1	0.10
3DLVEF, %	51.7±2.5	52.0±2.9	0.86	58.2±2.4	0.10
LV GLS, %	-16.1±0.9	-15.9±0.9	0.78	-17.8±0.9	0.92

Abbreviations: 3DLVEF – three-dimensional left ventricular (LV) ejection fraction; GLS – global longitudinal strain; LA – left atrial dimension; LAA – left atrial area; LVEDV – LV end-diastolic volume; LVESV – LV end-systolic volume; LVIDd – LV internal dimension at end diastole, LVIDs – LV internal dimension at end systole.
