# Supplementary material for: Early Effects of Modern Radiotherapy for Lung Cancer on Endothelial Damage and Myocardial Fibrosis: A Prospective Single-Center Study
Source: Int J Mol Sci. 2024 Jun 18;25(12):6705. doi: 10.3390/ijms25126705 (PMC11204135; doi:10.3390/ijms25126705)
Supplement: Supplementary file 1 [file ijms-25-06705-s001.zip › Supplementary Table S2.pdf]

Supplementary Table S2. Changes in right ventricular (RV) dimensions and parameters reflecting the RV systolic function.

Variable	Baseline	Immediately after treatment	p value	Three months after treatment	p value (vs baseline)
All patients (n=43)					
3DRVEF, %	52.0±7.0	51.1±6.6	0.34	50.4±6.0	0.56
RV FWLS, %	-22.5±5.6	-23.2±5.2	0.60	-22.2±5.9	0.27
RV GLS, %	-20.0±5.3	-19.0±3.6	0.09	-20.6±4.5	0.34
RV S', cm/s	13.7±3.3	13.4±2.2	0.56	13.7±2.8	0.79
TAPSE, mm	22.4±3.4	21.5±3.9	0.30	21.1±3.4	0.09
RVOT, mm	32.9±4.6	32.5±3.7	0.19	33.3±3.9	0.88
RVIT, mm	33.4±3.4	33.4±3.4	0.91	34.5±4.4	0.36
RA area, cm^2	14.1±3.3	14.4±2.8	0.43	14.5±3.4	0.75
Study group (n=23)					
3DRVEF, %	52.3±6.3	49.7±5.7	0.08	49.4±4.2	0.40
RV FWLS, %	-23.8±4.5	-22.9±5.3	0.38	-21.8±5.8	0.046
RV GLS, %	-21.1±4.0	-18.4±4.1	0.001	-19.1±4.3	0.016
RV S', cm/s	13.5±3.5	12.4±1.9	0.41	12.8±2.3	0.91
TAPSE, mm	22.1±3.3	20.3±3.2	0.021	20.8±3.3	0.051
RVOT, mm	33.2±5.4	32.9±2.2	0.07	33.1±3.4	0.09
RVIT, mm	34.3±3.1	33.7±3.3	0.24	36.0±3.2	0.18
RA area, cm^2	15.2±4.2	14.4±3.3	0.37	16.2±3.7	0.60
Control group (n=20)					
3DRVEF, %	51.8±7.9	52.5±7.2	0.73	51.7±7.9	0.92
RV FWLS, %	-21.2±6.4	-23.6±5.2	0.22	-22.8±6.4	0.82
RV GLS, %	-18.8±6.2	-19.8±2.9	0.80	-22.8±4.1	0.36
RV S', cm/s	14.0±3.0	14.5±2.0	0.81	14.8±3.1	0.81
TAPSE, mm	22.8±3.7	22.8±4.2	0.31	21.6±3.6	0.55
RVOT, mm	32.6±3.6	32.2±4.8	0.14	33.5±4.6	0.05
RVIT, mm	32.5±3.5	32.9±3.6	0.94	32.7±5.0	0.95
RA area, cm^2	13.0±1.5	14.3±2.2	0.03	12.5±1.5	0.74

Abbreviations: 3DRVEF – three-dimensional RV ejection fraction, RV FWLS – RV free wall longitudinal strain, RA – right atrium, RV S'– tissue Doppler-derived tricuspid annulus systolic velocity, RVIT – RV inflow tract, RVOT – RV outflow tract, TAPSE – tricuspid annular plane systolic excursion.
